# Intramedullary fixation of proximal humerus fractures: do locking bolts endanger the axillary nerve or the ascending branch of the anterior circumflex artery? A cadaveric study

**DOI:** 10.1186/1754-9493-2-33

**Published:** 2008-12-16

**Authors:** Stefaan Nijs, An Sermon, Paul Broos

**Affiliations:** 1 Department of Trauma Surgery, UZ Leuven, Herestraat 49, 3000 Leuven, Belgium

## Abstract

**Background:**

Proximal humerus fractures are one of the most common fractures. Intramedullary locked nailing is becoming a popular alternative treatment, especially for easier fracture patterns. Although axillary nerve injury has been reported, no study has compared the safety of the proximal locking options relative to the axillary nerve and the ascending branch of the anterior circumflex artery.

**Method:**

Six different commercially available proximal humeral nails were implanted in 30 shoulders of 18 cadavers. After fluoroscopically guided implantation the shoulders were carefully dissected and the distance between the locking screws, the axillary nerve and the ascending branch of the anterior circumflex artery was measured.

**Results:**

The course of the axillary nerve varies. A mean distance of 55.8 mm (SD = 5.3) between the lateral edge of the acromions and the axillary nerve at the middle of the humerus in a neutrally rotated position was observed. The minimum distance was 43.4 mm, the maximum 63.9 mm.

Bent nails with oblique head interlocking bolts appeared to be the most dangerous in relation to the axillary nerve. The two designs featuring such a bend and oblique bolt showed a mean distance of the locking screw to the axillary nerve of 1 mm and 2.7 mm respectively Sirus (Zimmer^®^) and (Stryker^®^) T2 PHN (Proximal Humeral Nail)).

Regarding the ascending branch of the anterior circumflex artery, there was no difference between the nails which have an anteroposterior locking option.

**Conclusion:**

It is of great importance for surgeons treating proximal humerus fractures to understand the relative risk of any procedure they perform. Since the designs of different nailing systems risk damaging the axillary nerve and ascending branch, blunt dissection, the use of protection sleeves during drilling and screw insertion, and individual risk evaluation prior to the use of a proximal humeral nail are advocated.

## Background

As proximal humerus fractures are amongst the most common fractures (third most common fracture) [1], there are many implants indicated for their treatment. Intramedullary implants are frequently used to treat A- and B-type fractures of the proximal humerus minimally invasively (even percutaneously). Due to the proximity of the axillary nerve, there is a potential risk of iatrogenic injury. The risk of these injuries was demonstrated in a previous publication from our group [2].

As the axillary nerve innervates the deltoid muscle, injury to this nerve dramatically affects the function of the shoulder[3]. With some implants there is an option to use an anteroposterior interlocking screw to stabilise fractures of the lesser tuberosity. This interlocking screw can endanger the ascending branch of the anterior humeral circumflex artery, the most important vessel in the vascularisation of the humeral head.

Little is known about the safety of the proximal interlocking bolts in relation to the abovementioned structures. Some studies document the relation between an interlocking bolts and the axillary nerve in specific types of nails used to treat shaft fractures [4-7]. One study investigates the safety of the spiral blade (Synthes, Bettlach, Switzerland) nail regarding iatrogenic axillary nerve damage[8]. However, there is no study which defines the relationship of interlocking bolts in other specific proximal humeral nails, and no study defines the safety of proximal interlocking options in relation to the ascending branch of the anterior humeral circumflex artery.

The purpose of our study was to define the safety of proximal interlocking options in six (6) commercially available nails, designed especially for the treatment of proximal humerus fractures.

## Method

Thirty shoulders from 18 preserved cadavers have were used. The upper extremities were intact and not separated from the trunk in order to preserve normal anatomy. The cadavers were installed and stabilised in beach chair position, with the lower arm resting on a support and the elbow flexed at 90°. Fluoroscopic access in two orthogonal planes was set up prior to surgery. Shoulders with a history of previous surgery, deformation due to marked omarthrosis or shoulders with a cuff tear were excluded from the study.

**Figure 1:**
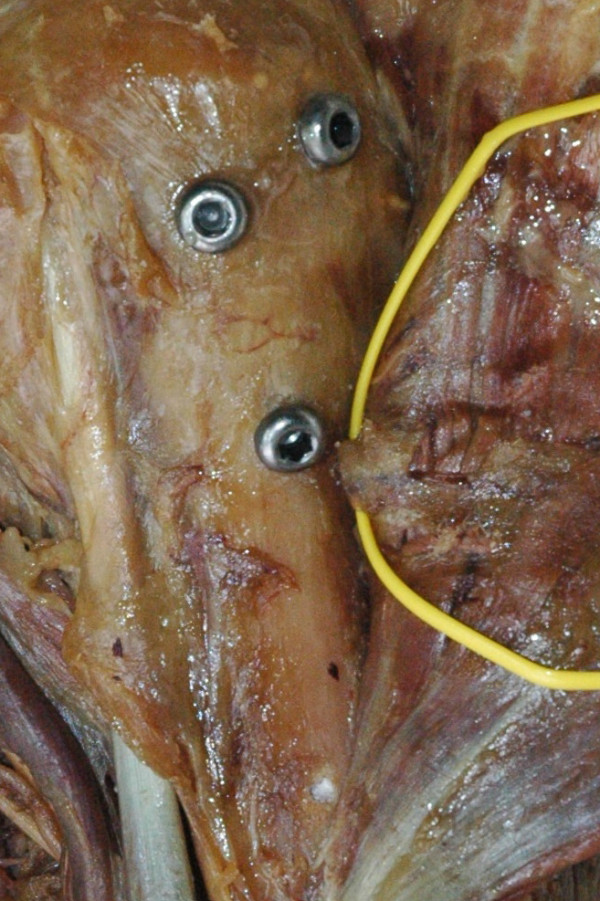
**Evaluation of the risk of iatrogenic injury to the axillary nerve using the Sirus Nail (Zimmer^®^)**. The minimal distance between the oblique inferior proximal locking bolt of the Sirus nail (Zimmer^®^) and the anterior branch of the axillary nerve puts this structure at risk during drilling and blunt insertion of the bolt.

**Figure 2:**
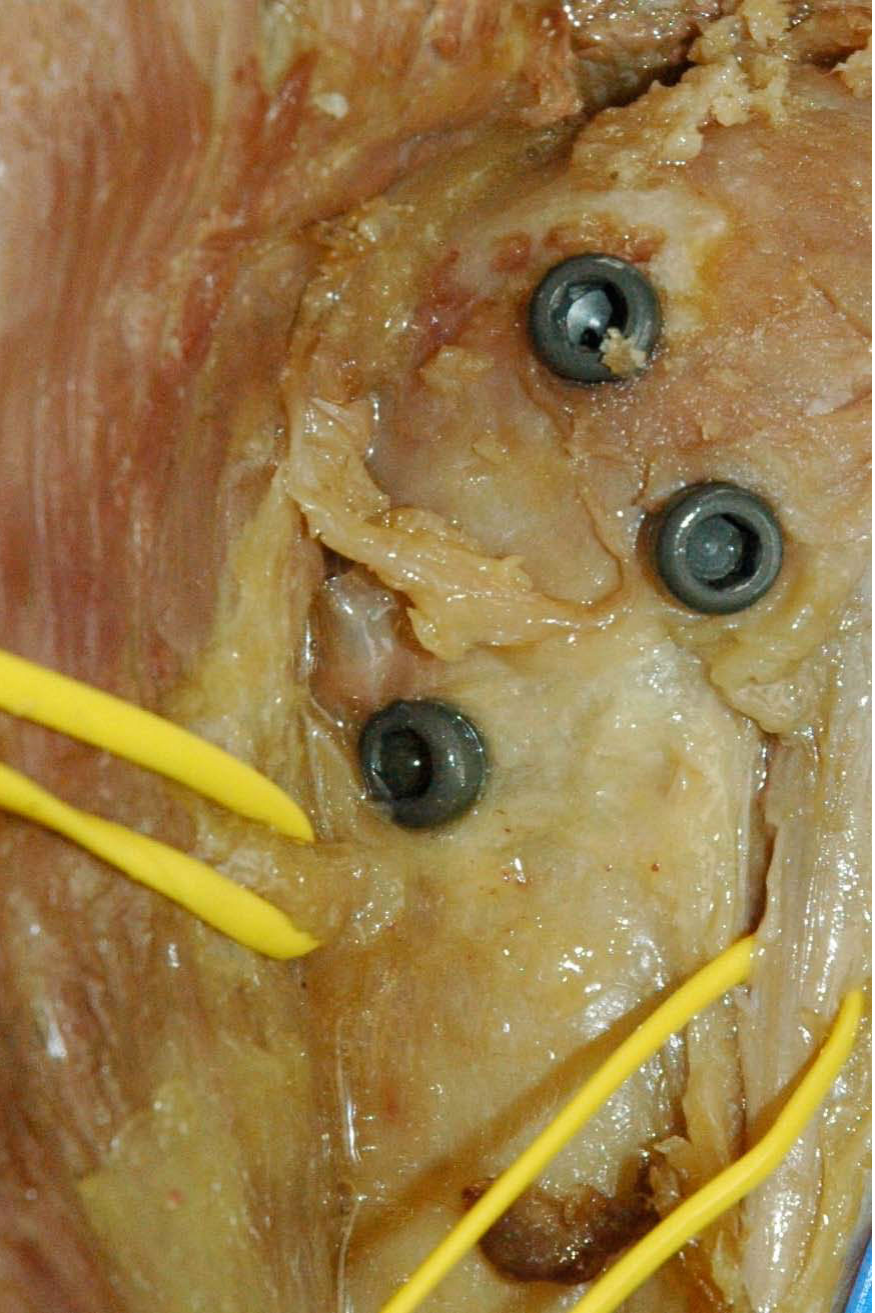
**Anatomic preparation of the proximal humerus after insertion of a T2 Nail (Stryker^®^)**. In the T2 Nail the limited distance between the inferior oblique proximal bolt and the anterior branch of the axillary nerve can put the nerve at risk during insertion of the bolt.

We tested the Synthes^® ^PHN (Proximal Humerus Nail) and Expert PHN, the Smith and Nephew^® ^Trigen PHN (straight version), the Stryker^® ^T2 PHN, the Aesculap^® ^Targon PH (Proximal Humerus) and the Zimmer^® ^Sirus Nail (figures 1 and 2). Descriptive data of the different nails tested can be found in Table 1. Exposure and nail insertion were performed using the surgical technique recommended by each nail manufacturer, using fluoroscopy in order to ensure exact nail positioning. All nails were implanted beneath the articular surface of the humeral head, ensuring central position of the central interlocking bolt in the lateral view, and symmetric positioning of the bolts in the humeral head in the AP view. The spiral blade in the Synthes^® ^PHN was positioned centrally in the head in the lateral view, and at the transition of the proximal to the distal third in the AP view. The oblique screw in the Sirus (Zimmer^®^) nail was positioned just above the calcar in the AP view, and centrally in the lateral view. The investigators performing the cadaveric surgeries (S.N. and A.S.) both have clinical experience with all of the nailing systems studied. The nails were distributed randomly amongst the shoulders, using a computerised randomisation protocol prior to preparation of the cadavers. It was ensured that the same type of nail was not implanted in both shoulders of the same cadaver.

**Table 1:** Characterstics of the six different implants used in this study.

**Nail**	**Bend**	**Length**	**Apex-bolt distance**	**Locking bolt orientation**
Synthes PHN	6°	150 mm	18 mm	90°

Synthes EPHN	6°	150 mm	20 mm	90°

Targon PH	0°	150 mm	26 mm	90°

Stryker T2-PHN	6°	150 mm	30 mm	105°

S&N Trigen PH	0°	160 mm	32 mm	90°

Zimmer Sirus	9°	120 mm	37 mm	120°

An anterior delta split incision of 2 cm was made, directly anterior to the acromial edge. A K-wire was positioned under fluoroscopic guidance to locate the exact entry point. The proximal humerus was opened using an awl. The nail was introduced, and its exact position was verified under fluoroscopic control. For the straight nails the entry point was on the highest point of the humeral head in the AP view, and centrally on the head in the lateral view. For the bent nails the entry point was on the transition of the humeral head to the greater tuberosity in the AP view and again centrally on the head in the lateral view. Special care was taken to implant the nail at the exact height. The proximal and distal locking bolts were introduced percutaneously using the nails-specific guide, again under fluoroscopic control.

Following bolt insertion, the anterior deltoid was dissected from the acromion and retracted laterally. The axillary nerve, the acromion, the ascending branch of the anterior circumflex artery and the locking screws were identified, taking care not to disturb the natural position of the anatomical structures. One investigator measured the following distances, using calipers:

• Acromion-axillary nerve

• Locking bolts-axillary nerve

• Acromion locking bolts

• Locking bolts-ascending branch anterior circumflex artery

When measuring the distance to a bolt, the distance to the edge of the bolt-head was measured. Every measurement was repeated three times (non-consecutively), taking the mean to minimise error. To address the varied positions of the axillary nerve we also compared the distance of the bolts to the average position of the axillary nerve based upon our measurements in 30 cadaveric shoulders.

## Results

The position of the axillary nerve varies. On average a distance of 55.8 mm (SD = 5.3) was recorded between the lateral edge of the acromions and the axillary nerve at the middle of the humerus in a neutrally rotated position. The minimum distance was 43.4 mm, the maximum 63.9 mm.

The results of the exact measurements are summarised in Table 2. The Sirus nail (Zimmer^®^) proved to be the most dangerous to the axillary nerve. In 4/5 cadavers the axillary nerve was damaged during insertion of the oblique interlocking bolt. The T2 PHN (Stryker^®^) proved to be the second most dangerous. With a mean distance of 2.7 mm between the third, most oblique bolt and the axillary nerve, it most certainly puts the nerve at risk during insertion off the bolt. The mean distance between the spiral blade of the EPHN (Synthes^®^) and the nerve was on average 19.2 mm. However, the oblique bolt and the second transverse bolt in the PHN were only 7.5 and 6.4 mm (mean values) from the nerve respectively. The safest proved to be the straight nails: Targon PH (Aesculap^®^) and Trigen PHN (Smith and Nephew^®^) with mean distances of 136 mm and 10 mm respectively.

**Table 2:** Average distance of the (closest) locking bolt to the axillary nerve and the ascending branch of the anterior circumflex artery.

**Nail**	**bolt-axillaris distance (mean)**	**SD**	**bolt-ascending branch distance (mean)**	**SD**
Synthes PHN	7.48 mm	3.5	19.2 mm	6.0

Synthes EPHN	6.4 mm	5,1	14.2 mm	3.0

Targon PH	13.6 mm	8,1	5.8 mm	3.1

Stryker T2-PHN	2.7 mm	1,1	8 mm	4.3

S&N Trigen PH	10 mm	2,7	8.1 mm	2.1

Zimmer Sirus	1 mm	2	14.1 mm	3.9

Regarding the ascending branch of the anterior circumflex artery, the designs with an anteroposterior interlocking bolt are, of course, of most interest. With mean distances of 8 mm, 81 mm and 58 mm on average, there is no significant difference between the T2 PHN (Stryker^®^), Trigen PH (Smith and Nephew^®^) and Targon PH (Aesculap^®^).

Because there was a lot of variance in the position of the axillary nerve, we also compared the mean distances of the interlocking bolts to the acromions in relation to an overall average position of the axillary nerve relative to the acromion (based upon 30 cadaveric shoulders). The results of this calculation are presented in Table 3. This gives a more nuanced picture, but again the nails with an oblique interlocking bolt (T2 PHN (Stryker^®^) and Sirus (Zimmer^®^) have the worst scores. The additional oblique and transverse bolts in the PHN and EPHN (Synthes) respectively, are about 8 mm from the axillary nerve (mean values). Since their contribution to stability is not proven, it may be safer only to use only the spiral blade with a mean distance of 12.1 mm to the axillary nerve.

**Table 3:** Mean distance of the (closest) locking bolt to the theoretical average position of the axillary nerve

**Nail**	**bolt-axillaris distance**	**SD**
Synthes PHN	8.6 mm	3.4

Synthes EPHN	8.2 mm	6.0

Targon PH	15.7 mm	2.1

Stryker T2-PHN	2.7 mm	1.9

S&N Trigen PH	6.7 mm	5.2

Zimmer Sirus	5.0 mm	2.3

## Discussion

The axillary nerve originates from the posterior cord of the brachial plexus and runs along the subscapular muscle. At the glenoid neck it runs posteriorly, and passes through the quadrilateral space. In the majority of specimen, the nerve splits in a posterior trunk and an anterior trunk just anterior to the origin of the long head of the triceps. However, according to a recent study of Loukas[9], the nerve only splits in the deltoid muscle in 35% of specimens. The posterior trunk branches off to the teres minor and to the posterior part of the deltoid muscle. It continues as the superior lateral brachial cutaneous branch innervating the skin over the deltoid muscle. The anterior trunk runs on the deep subfascial surface and within the deltoid muscle at the level of the surgical neck of the humerus, where it branches off and supplies the acromial and clavicular part of the deltoid muscle. The anterior branch also sends branches to the joint capsule. As previously mentioned, the position of the axillary nerve at the lateral aspect of the shoulder does vary, and is therefore susceptible to injury during surgical procedures splitting the deltoid.

In our study the distance to the lateral edge of the acromion orthogonal to the centre of the lateral humerus projection, with the arm in a neutral position was 55.8 (SD = 5.3 mm). The minimum distance measured was 43.4 mm and the maximum 63.9 mm. These results are consistent with previous reports in the literature. Bono reported a mean distance between the top of the humeral head and the axillary nerve to be 61 +/- 7 mm (range 45 to 69 mm). The work of Cetik[10] showed the mean anterior acromio-axillary distance was 60.8 mm and the mean posterior distance 48.7 mm. Kamineni [11]did recorded a mean distance posteriorly of 57 mm (range 35 to 70 mm) and anteriorly of 51 mm (range 35–85 mm). The variability however makes it difficult to compare the distance between interlocking screws and the nerve in a limited number of implantations. Therefore, we not only detail the distances measured directly, but also relative to an average position, based upon measurement in all 30 cadaveicr shoulders.

The average distance of the nearest interlocking bolt to the axillary nerve varied significantly in our series. When a minimum distance of this locking bolt to the nerve of 5 mm is considered to be safe, both the T2 (Stryker^®^) and the Sirus (Zimmer^®^) nail fail to reach these safety margins, as well in direct measurement as in distance relative to the "average" axillary nerve. Other designs tend to be safer, especially when the secondary bolt is not used in the PHN (Synthes^®^). However, due to the varied position, all implants could endanger the axillary nerve. A (iatrogenic) lesion of the axillary nerve seriously compromises the function of the shoulder. Even a transient dysfunction of the deltoid can jeopardise the ability to cope with normal post-operative active rehabilitation, and as such increase the risk of (permanent) restricted shoulder motion.

The relative position of the ascending branch of the anterior circumflex artery is more consistent, being in the bicipital groove. However as the size of the proximal humerus varies according to patients' height and build, this present another variable. Iatrogenic damage to the ascending branch of the circumflex artery (the main nutrient vessel to the humeral head) can promote avascular necrosis. Anteroposterior interlocking bolts can endanger the ascending branch. On average the bolts were more than 5 mm from the artery, but in 4/15 cases where an anteroposterior bolt was implanted, there was a mean distance of less than 5 mm from the artery.

We determined the position of the bolt relative to an intact proximal humerus with unchanged position of the axillary nerve and ascending branch. Of course, fracture haematoma and non-anatomical reposition can alter the position of these structures relative to the bone. This only increases the risk of iatrogenic injury of the axillary nerve and the ascending branch of the anterior circumflex artery.

## Conclusion

It is of great importance for surgeons treating proximal humerus fractures to understand the relative risk of any procedure they perform. Since the designs of different nailing systems risk damaging the axillary nerve and ascending branch, blunt dissection, the use of protection sleeves during drilling and screw insertion, and individual risk evaluation prior to the use of a proximal humeral nail are advocated.

## Competing interests

None of the authors has any direct financial interests in any of the implants described in the article or in any of the manufacturers. All of the companies provided us with the implants used in this study free of charge. All of the companies mentioned in the article have sponsored the research fund of the authors' Department during the last five years. No direct financial sponsorship of this study has been received from any company.

## Authors' contributions

SN conceived, designed and coordinated the study and drafted the manuscript. AS performed part of the implantations. PB helped to draft the manuscript. All authors read and approved the final manuscript.
